# *N*-(2,5-Dichloro­phen­yl)succinamic acid

**DOI:** 10.1107/S1600536811028297

**Published:** 2011-07-23

**Authors:** B. S. Saraswathi, Sabine Foro, B. Thimme Gowda

**Affiliations:** a Department of Chemistry, Mangalore University, Mangalagangotri 574 199, Mangalore, India; b Institute of Materials Science, Darmstadt University of Technology, Petersenstrasse 23, D-64287 Darmstadt, Germany

## Abstract

In the title compound, C_10_H_9_Cl_2_NO_3_, the conformation of the N—H bond in the amide segment is *syn* with respect to the *ortho*-Cl atom and *anti* to the *meta*-Cl atom of the benzene ring. In the crystal, inter­molecular O—H⋯O and N—H⋯O hydrogen bonds pack the mol­ecules into two types of chains along the *a* and *b* axes, respectively, leading to an overall sheet structure. The acid group in the side chain is disordered and was refined using a split model with site-occupation factors of 0.60:0.40.

## Related literature

For our studies of the effects of substituents on the structures and other aspects of *N*-(ar­yl)-amides, see: Bhat & Gowda (2000 ▶); Gowda *et al.* (2007 ▶); Saraswathi *et al.* (2011**a* ▶,b* ▶), on *N*-(ar­yl)-methane­sulfonamides, see: Jayalakshmi & Gowda (2004 ▶) and on *N*-chloro-aryl­sulfonamides, see: Gowda *et al.* (2003 ▶). For the modes of inter­linking carb­oxy­lic acids by hydrogen bonds, see: Leiserowitz (1976 ▶). For the packing of mol­ecules involving dimeric hydrogen-bonding associations of each carboxyl group with a centrosymmetrically related neighbor, see: Jagannathan *et al.* (1994 ▶).
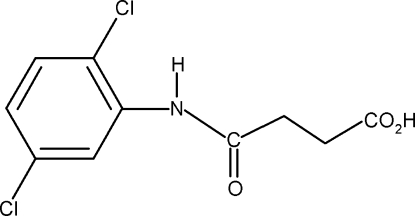


## Experimental

### 

#### Crystal data

C_10_H_9_Cl_2_NO_3_
*M* *_r_* = 262.08
Monoclinic, 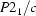

*a* = 5.726 (1) Å
*b* = 4.787 (1) Å
*c* = 41.583 (6) Å
β = 91.93 (2)°
*V* = 1139.2 (4) Å^3^
*Z* = 4
Mo *K*α radiation
μ = 0.56 mm^−1^
*T* = 293 K
0.44 × 0.16 × 0.09 mm

#### Data collection

Oxford Diffraction Xcalibur diffractometer with a Sapphire CCD detector
Absorption correction: multi-scan (*CrysAlis RED*; Oxford Diffraction, 2009 ▶) *T*_min_ = 0.791, *T*_max_ = 0.951
3375 measured reflections
2046 independent reflections
1552 reflections with *I* > 2σ(*I*)
*R*_int_ = 0.022

#### Refinement

*R*[*F* ^2^ > 2σ(*F* ^2^)] = 0.077
*wR*(*F* ^2^) = 0.171
*S* = 1.14
2046 reflections
185 parameters
54 restraints
H atoms treated by a mixture of independent and constrained refinement
Δρ_max_ = 0.75 e Å^−3^
Δρ_min_ = −0.41 e Å^−3^

### 

Data collection: *CrysAlis CCD* (Oxford Diffraction, 2009 ▶); cell refinement: *CrysAlis RED* (Oxford Diffraction, 2009 ▶); data reduction: *CrysAlis RED*; program(s) used to solve structure: *SHELXS97* (Sheldrick, 2008 ▶); program(s) used to refine structure: *SHELXL97* (Sheldrick, 2008 ▶); molecular graphics: *PLATON* (Spek, 2009 ▶); software used to prepare material for publication: *SHELXL97*.

## Supplementary Material

Crystal structure: contains datablock(s) I, global. DOI: 10.1107/S1600536811028297/vm2110sup1.cif

Structure factors: contains datablock(s) I. DOI: 10.1107/S1600536811028297/vm2110Isup2.hkl

Supplementary material file. DOI: 10.1107/S1600536811028297/vm2110Isup3.cml

Additional supplementary materials:  crystallographic information; 3D view; checkCIF report

## Figures and Tables

**Table 1:** Hydrogen-bond geometry (Å, °)

*D*—H⋯*A*	*D*—H	H⋯*A*	*D*⋯*A*	*D*—H⋯*A*
O2*A*—H2*A*⋯O3*A*^i^	0.82	1.90	2.687 (15)	162
O2*B*—H2*B*⋯O3*B*^i^	0.82	1.90	2.64 (2)	150
N1—H1*N*⋯O1^ii^	0.85 (2)	2.07 (2)	2.901 (6)	167 (5)

Symmetry codes: (i) 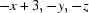; (ii) 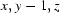.

**Fig. 1.**
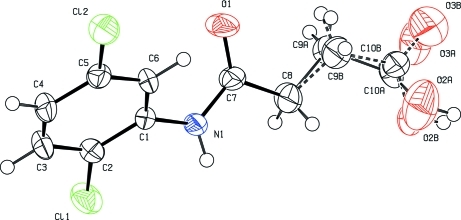
Molecular structure of the title compound, showing the atom labelling scheme and with displacement ellipsoids drawn at the 50% probability level.

**Fig. 2.**
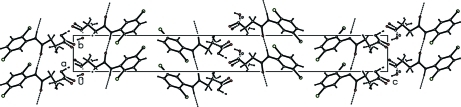
Molecular packing of the title compound with hydrogen bonding shown as dashed lines.

## supplementary crystallographic information

### Comment

The amide and sulfonamide moieties are important constituents of many biologically important compounds. As a part of our studies on the effects of substituents on the structures and other aspects of this class of compounds (Bhat & Gowda, 2000; Gowda *et al.*, 2003, 2007; Jayalakshmi & Gowda, 2004; Saraswathi *et al.*, 2011**a*,b*), in the present work, the crystal structure of *N*-(2,5-dichlorophenyl)-succinamic acid (I) has been determined (Fig. 1). The conformation of the N—H bond in the amide segment is *syn* to the *ortho*–chloro atom and *anti* to the *meta*–chloro atom of the benzene ring, similar to the *syn* conformation observed between the amide hydrogen and the *ortho*- methyl group and *anti* conformation between the amide hydrogen and the *meta*-methyl group in the benzene ring of *N*-(2,5-dimethylphenyl)-succinamic acid monohydrate (II) (Saraswathi *et al.*, 2011*a*).

Further, the conformations of the amide oxygen and the carboxyl oxygen of the acid segment are *syn* to each other. But the conformation of the amide C═O is *anti* to the H atoms on the adjacent –CH_2_ group, while the carboxyl C═O is *syn* to the H atoms on the adjacent –CH_2_ group.

The C═O and O—H bonds of the acid group are in *syn* position to each other, similar to that observed in (II) and in *N*-(2,6-dichlorophenyl)-succinamic acid (Saraswathi *et al.*, 2011*b*).

The intermolecular O—H···O and N—H···O hydrogen bonds, along *a*- and *b*-axes, respectively, pack the molecules into infinite chains in the structure (Table 1, Fig.2).

The modes of interlinking carboxylic acids by hydrogen bonds is described elsewhere (Leiserowitz, 1976). The packing of molecules involving dimeric hydrogen bonded association of each carboxyl group with a centrosymmetrically related neighbor has also been observed (Jagannathan *et al.*, 1994).

### Experimental

The solution of succinic anhydride (0.01 mole) in toluene (25 ml) was treated dropwise with the solution of 2,5-dichloroaniline (0.01 mole) also in toluene (20 ml) with constant stirring. The resulting mixture was stirred for about one hour and set aside for an additional hour at room temperature for completion of the reaction. The mixture was then treated with dilute hydrochloric acid to remove the unreacted 2,5-dichloroaniline. The resultant title compound was filtered under suction and washed thoroughly with water to remove the unreacted succinic anhydride and succinic acid. It was recrystallized to constant melting point from ethanol. The purity of the compound was checked and characterized by its infrared and NMR spectra.

Needle like colorless single crystals used in X-ray diffraction studies were grown in ethanolic solution by slow evaporation at room temperature.

### Refinement

The H atom of the NH group was located in a difference map and its position refined with N—H = 0.86 (2) Å. The other H atoms were positioned with idealized geometry using a riding model with the aromatic C—H = 0.93 Å, methylene C—H = 0.97 Å and O—H = 0.82 Å. All H atoms were refined with isotropic displacement parameters (set to 1.2 times of the *U*_eq_ of the parent atom).

The atoms C9, C10, O2 and O3 are disordered and were refind using a split model. The corresponding site-occupation factors were fixed to 0.60:0.40 and their corresponding bond distances in the disordered groups were restrained to be equal. The *U*_eq_ of these atoms were restrained to approximate isotropic behavoir.

### Crystal data

**Table:**

C_10_H_9_Cl_2_NO_3_	*F*(000) = 536
*M**_r_* = 262.08	*D*_x_ = 1.528 Mg m^−^^3^
Monoclinic, *P*2_1_/*c*	Mo *K*α radiation, λ = 0.71073 Å
Hall symbol: -P 2ybc	Cell parameters from 987 reflections
*a* = 5.726 (1) Å	θ = 2.9–27.7°
*b* = 4.787 (1) Å	µ = 0.56 mm^−^^1^
*c* = 41.583 (6) Å	*T* = 293 K
β = 91.93 (2)°	Needle, colourless
*V* = 1139.2 (4) Å^3^	0.44 × 0.16 × 0.09 mm
*Z* = 4	

### Data collection

**Table:**

Oxford Diffraction Xcalibur diffractometer with a Sapphire CCD detector	2046 independent reflections
Radiation source: fine-focus sealed tube	1552 reflections with *I* > 2σ(*I*)
graphite	*R*_int_ = 0.022
Rotation method data acquisition using ω scans	θ_max_ = 25.3°, θ_min_ = 2.9°
Absorption correction: multi-scan (*CrysAlis RED*; Oxford Diffraction, 2009)	*h* = −5→6
*T*_min_ = 0.791, *T*_max_ = 0.951	*k* = −5→2
3375 measured reflections	*l* = −50→37

### Refinement

**Table:**

Refinement on *F*^2^	Primary atom site location: structure-invariant direct methods
Least-squares matrix: full	Secondary atom site location: difference Fourier map
*R*[*F*^2^ > 2σ(*F*^2^)] = 0.077	Hydrogen site location: inferred from neighbouring sites
*wR*(*F*^2^) = 0.171	H atoms treated by a mixture of independent and constrained refinement
*S* = 1.14	*w* = 1/[σ^2^(*F*_o_^2^) + (0.0436*P*)^2^ + 4.544*P*] where *P* = (*F*_o_^2^ + 2*F*_c_^2^)/3
2046 reflections	(Δ/σ)_max_ = 0.048
185 parameters	Δρ_max_ = 0.75 e Å^−^^3^
54 restraints	Δρ_min_ = −0.41 e Å^−^^3^

### Special details

**Table:**

Geometry. All e.s.d.'s (except the e.s.d. in the dihedral angle between two l.s. planes) are estimated using the full covariance matrix. The cell e.s.d.'s are taken into account individually in the estimation of e.s.d.'s in distances, angles and torsion angles; correlations between e.s.d.'s in cell parameters are only used when they are defined by crystal symmetry. An approximate (isotropic) treatment of cell e.s.d.'s is used for estimating e.s.d.'s involving l.s. planes.
Refinement. Refinement of *F*^2^ against ALL reflections. The weighted *R*-factor *wR* and goodness of fit *S* are based on *F*^2^, conventional *R*-factors *R* are based on *F*, with *F* set to zero for negative *F*^2^. The threshold expression of *F*^2^ > σ(*F*^2^) is used only for calculating *R*-factors(gt) *etc*. and is not relevant to the choice of reflections for refinement. *R*-factors based on *F*^2^ are statistically about twice as large as those based on *F*, and *R*- factors based on ALL data will be even larger.

### Fractional atomic coordinates and isotropic or equivalent isotropic displacement parameters (Å^2^)

**Table:**

	*x*	*y*	*z*	*U*_iso_*/*U*_eq_	Occ. (<1)
Cl1	0.3416 (2)	−0.1995 (3)	0.13931 (4)	0.0513 (4)	
Cl2	0.8927 (3)	0.6671 (3)	0.22318 (3)	0.0460 (4)	
O1	0.8848 (9)	0.5091 (8)	0.10285 (10)	0.0599 (13)	
O2A	1.4182 (19)	−0.004 (3)	0.0382 (2)	0.088 (4)	0.60
H2A	1.4940	−0.0747	0.0239	0.106*	0.60
O2B	1.325 (3)	−0.072 (3)	0.0293 (4)	0.061 (4)	0.40
H2B	1.3972	−0.1171	0.0134	0.074*	0.40
O3A	1.259 (2)	0.248 (3)	−0.0021 (3)	0.082 (4)	0.60
O3B	1.357 (4)	0.322 (4)	0.0053 (5)	0.096 (7)	0.40
N1	0.7824 (8)	0.0816 (9)	0.12040 (10)	0.0342 (10)	
H1N	0.791 (9)	−0.091 (5)	0.1164 (13)	0.041*	
C1	0.6994 (8)	0.1621 (10)	0.15067 (11)	0.0296 (11)	
C2	0.4967 (9)	0.0438 (11)	0.16233 (12)	0.0342 (12)	
C3	0.4144 (9)	0.1202 (12)	0.19205 (13)	0.0402 (13)	
H3	0.2780	0.0402	0.1994	0.048*	
C4	0.5332 (9)	0.3132 (12)	0.21069 (13)	0.0412 (13)	
H4	0.4777	0.3662	0.2305	0.049*	
C5	0.7367 (9)	0.4276 (11)	0.19951 (12)	0.0334 (12)	
C6	0.8201 (8)	0.3543 (11)	0.17001 (11)	0.0321 (12)	
H6	0.9576	0.4337	0.1630	0.039*	
C7	0.8798 (10)	0.2569 (11)	0.09932 (13)	0.0369 (13)	
C8	0.9872 (12)	0.1158 (13)	0.07101 (14)	0.0516 (16)	
H8A	1.1120	−0.0047	0.0791	0.062*	
H8B	0.8695	−0.0031	0.0608	0.062*	
C9A	1.082 (2)	0.297 (3)	0.0464 (2)	0.044 (3)	0.60
H9A	1.1601	0.4541	0.0568	0.053*	0.60
H9B	0.9545	0.3688	0.0329	0.053*	0.60
C9B	1.172 (3)	0.293 (5)	0.0582 (5)	0.058 (5)	0.40
H9C	1.2859	0.3277	0.0756	0.069*	0.40
H9D	1.1028	0.4709	0.0523	0.069*	0.40
C10A	1.253 (3)	0.145 (3)	0.0256 (4)	0.057 (4)	0.60
C10B	1.302 (4)	0.187 (4)	0.0298 (5)	0.043 (6)	0.40

### Atomic displacement parameters (Å^2^)

**Table:**

	*U*^11^	*U*^22^	*U*^33^	*U*^12^	*U*^13^	*U*^23^
Cl1	0.0426 (8)	0.0500 (9)	0.0613 (9)	−0.0162 (7)	0.0020 (7)	−0.0020 (8)
Cl2	0.0553 (9)	0.0455 (8)	0.0376 (7)	−0.0051 (7)	0.0053 (6)	−0.0069 (7)
O1	0.106 (4)	0.026 (2)	0.050 (3)	−0.007 (2)	0.040 (2)	−0.0065 (19)
O2A	0.092 (7)	0.118 (8)	0.055 (5)	0.027 (6)	0.018 (5)	−0.009 (5)
O2B	0.068 (6)	0.056 (5)	0.062 (6)	0.002 (4)	0.031 (4)	−0.004 (4)
O3A	0.075 (6)	0.109 (8)	0.065 (6)	0.019 (6)	0.017 (5)	−0.005 (6)
O3B	0.110 (10)	0.081 (9)	0.099 (10)	0.001 (8)	0.038 (8)	0.009 (8)
N1	0.044 (2)	0.024 (2)	0.037 (2)	−0.004 (2)	0.0166 (19)	−0.004 (2)
C1	0.029 (2)	0.025 (3)	0.035 (3)	0.003 (2)	0.011 (2)	0.001 (2)
C2	0.031 (3)	0.030 (3)	0.042 (3)	−0.003 (2)	0.005 (2)	0.001 (2)
C3	0.032 (3)	0.045 (3)	0.044 (3)	−0.003 (3)	0.016 (2)	0.008 (3)
C4	0.042 (3)	0.046 (3)	0.037 (3)	0.004 (3)	0.020 (2)	0.005 (3)
C5	0.037 (3)	0.031 (3)	0.032 (3)	0.004 (2)	0.007 (2)	0.002 (2)
C6	0.028 (3)	0.033 (3)	0.037 (3)	−0.001 (2)	0.013 (2)	0.005 (2)
C7	0.050 (3)	0.026 (3)	0.035 (3)	−0.002 (2)	0.012 (2)	−0.006 (2)
C8	0.069 (4)	0.043 (3)	0.045 (3)	−0.007 (3)	0.027 (3)	−0.012 (3)
C9A	0.062 (6)	0.039 (5)	0.032 (5)	0.007 (5)	0.013 (4)	0.002 (5)
C9B	0.064 (9)	0.050 (8)	0.061 (9)	−0.001 (8)	0.018 (7)	−0.011 (8)
C10A	0.061 (7)	0.041 (7)	0.073 (8)	−0.009 (5)	0.043 (6)	0.002 (6)
C10B	0.048 (8)	0.037 (8)	0.045 (7)	−0.001 (5)	0.009 (4)	−0.004 (5)

### Geometric parameters (Å, °)

**Table:**

Cl1—C2	1.733 (5)	C3—H3	0.9300
Cl2—C5	1.739 (5)	C4—C5	1.382 (7)
O1—C7	1.217 (6)	C4—H4	0.9300
O2A—C10A	1.282 (15)	C5—C6	1.377 (7)
O2A—H2A	0.8200	C6—H6	0.9300
O2B—C10B	1.246 (17)	C7—C8	1.507 (7)
O2B—H2B	0.8200	C8—C9A	1.458 (12)
O3A—C10A	1.254 (12)	C8—C9B	1.47 (2)
O3B—C10B	1.256 (15)	C8—H8A	0.9700
N1—C7	1.348 (7)	C8—H8B	0.9700
N1—C1	1.414 (6)	C9A—C10A	1.516 (12)
N1—H1N	0.85 (2)	C9A—H9A	0.9700
C1—C6	1.391 (7)	C9A—H9B	0.9700
C1—C2	1.393 (7)	C9B—C10B	1.505 (16)
C2—C3	1.386 (7)	C9B—H9C	0.9700
C3—C4	1.372 (8)	C9B—H9D	0.9700
			
C10A—O2A—H2A	109.5	C9A—C8—C7	117.0 (6)
C10B—O2B—H2B	109.5	C9B—C8—C7	110.1 (8)
C7—N1—C1	124.6 (4)	C9A—C8—H8A	108.1
C7—N1—H1N	117 (4)	C9B—C8—H8A	86.4
C1—N1—H1N	118 (4)	C7—C8—H8A	108.1
C6—C1—C2	118.1 (4)	C9A—C8—H8B	108.1
C6—C1—N1	121.3 (4)	C9B—C8—H8B	132.7
C2—C1—N1	120.5 (4)	C7—C8—H8B	108.1
C3—C2—C1	121.0 (5)	H8A—C8—H8B	107.3
C3—C2—Cl1	119.1 (4)	C8—C9A—C10A	112.3 (10)
C1—C2—Cl1	119.9 (4)	C8—C9A—H9A	109.1
C4—C3—C2	120.3 (5)	C10A—C9A—H9A	109.1
C4—C3—H3	119.8	C8—C9A—H9B	109.1
C2—C3—H3	119.8	C10A—C9A—H9B	109.1
C3—C4—C5	119.0 (5)	H9A—C9A—H9B	107.9
C3—C4—H4	120.5	C8—C9B—C10B	118.2 (17)
C5—C4—H4	120.5	C8—C9B—H9C	107.8
C6—C5—C4	121.4 (5)	C10B—C9B—H9C	107.7
C6—C5—Cl2	119.1 (4)	C8—C9B—H9D	107.8
C4—C5—Cl2	119.6 (4)	C10B—C9B—H9D	107.8
C5—C6—C1	120.2 (4)	H9C—C9B—H9D	107.1
C5—C6—H6	119.9	O3A—C10A—O2A	123.5 (12)
C1—C6—H6	119.9	O3A—C10A—C9A	112.0 (11)
O1—C7—N1	123.3 (5)	O2A—C10A—C9A	121.0 (14)
O1—C7—C8	122.0 (5)	O2B—C10B—O3B	117.8 (19)
N1—C7—C8	114.7 (5)	O2B—C10B—C9B	113.7 (18)
C9A—C8—C9B	27.7 (8)	O3B—C10B—C9B	127.6 (18)
			
C7—N1—C1—C6	−39.9 (8)	C1—N1—C7—O1	−7.7 (9)
C7—N1—C1—C2	142.0 (5)	C1—N1—C7—C8	171.2 (5)
C6—C1—C2—C3	1.5 (8)	O1—C7—C8—C9A	−4.8 (11)
N1—C1—C2—C3	179.7 (5)	N1—C7—C8—C9A	176.2 (7)
C6—C1—C2—Cl1	−179.4 (4)	O1—C7—C8—C9B	24.5 (13)
N1—C1—C2—Cl1	−1.2 (7)	N1—C7—C8—C9B	−154.4 (10)
C1—C2—C3—C4	−0.6 (8)	C9B—C8—C9A—C10A	79 (2)
Cl1—C2—C3—C4	−179.7 (4)	C7—C8—C9A—C10A	160.8 (11)
C2—C3—C4—C5	−0.6 (8)	C9A—C8—C9B—C10B	−70 (2)
C3—C4—C5—C6	0.8 (8)	C7—C8—C9B—C10B	179.8 (15)
C3—C4—C5—Cl2	−178.7 (4)	C8—C9A—C10A—O3A	152.1 (15)
C4—C5—C6—C1	0.1 (8)	C8—C9A—C10A—O2A	−48 (2)
Cl2—C5—C6—C1	179.7 (4)	C8—C9B—C10B—O2B	−33 (3)
C2—C1—C6—C5	−1.3 (7)	C8—C9B—C10B—O3B	136 (3)
N1—C1—C6—C5	−179.4 (5)		

### Hydrogen-bond geometry (Å, °)

**Table:**

*D*—H···*A*	*D*—H	H···*A*	*D*···*A*	*D*—H···*A*
O2A—H2A···O3A^i^	0.82	1.90	2.687 (15)	162.
O2B—H2B···O3B^i^	0.82	1.90	2.64 (2)	150.
N1—H1N···O1^ii^	0.85 (2)	2.07 (2)	2.901 (6)	167 (5)

Symmetry codes: (i) −*x*+3, −*y*, −*z*; (ii) *x*, *y*−1, *z*.
